# Fulminant Macrolide-Resistant Mycoplasma pneumoniae Pneumonia in a Young Adult: A Case Report

**DOI:** 10.7759/cureus.89595

**Published:** 2025-08-08

**Authors:** Yusuke Shimada, Shigeru Koba, Tsubasa Onishi, Jun Kataoka

**Affiliations:** 1 Department of Critical Care Medicine, Nerima Hikarigaoka Hospital, Tokyo, JPN; 2 Department of Infectious Disease, Nerima Hikarigaoka Hospital, Tokyo, JPN

**Keywords:** ards (acute respiratory distress syndrome), diagnosis and treatment strategy, infectious disease diagnosis, macrolide-resistant mycoplasma pneumoniae, mycoplasma pneumoniae pneumonia

## Abstract

*Mycoplasma pneumoniae* commonly causes community-acquired pneumonia (CAP) in young adults, but it rarely leads to acute respiratory distress syndrome (ARDS). Macrolides are commonly used as the first-line treatment for *M. pneumoniae* pneumonia; however, the incidence of macrolide-resistant *M. pneumoniae* (MRMP) has increased, particularly in East Asia. There are few case reports of severe ARDS in adults caused by MRMP. Here, we present the case of a 27-year-old woman with severe MRMP pneumonia who required mechanical ventilation. Despite repeated negative rapid antigen tests for *M. pneumoniae*, the patient was suspected of having MRMP pneumonia based on the Japanese Respiratory Society (JRS) scoring system and bilateral pneumonia resistant to amoxicillin/clavulanate and azithromycin, which led to the administration of levofloxacin infusion. Hydrocortisone was administered as treatment for severe CAP. Lung-protective ventilation and prone positioning were implemented to manage severe ARDS. The patient’s condition improved rapidly, with extubation on day 4 and discharge without complications on day 10. Previously, patients were treated based on positive test results. In this case, despite negative repeated rapid antigen tests and prior macrolide treatment, the probability of *M. pneumoniae* was evaluated, and treatment for MRMP was promptly initiated upon ICU admission. The diagnosis and treatment strategy resulted in early improvement. This case highlights the diagnostic approach for MRMP and the clinical course of severe ARDS associated with MRMP.

## Introduction

*Mycoplasma pneumoniae* is a common cause of community-acquired pneumonia (CAP) in young adults [1]. In most cases, *M. pneumoniae* infections are self-limiting and resolve without the need for antibiotics [2]. In rare cases, patients develop fulminant conditions, with severe acute respiratory distress syndrome (ARDS) occurring in 0.5-2% of all cases [3]. A previous study found that only 1.3% of intensive care unit (ICU) patients with severe CAP had *M. pneumoniae* [4]. Consequently, accurately diagnosing *M. pneumoniae* pneumonia in patients with ARDS is challenging. Loop-mediated isothermal amplification (LAMP) or quenching-probe polymerase chain reaction (QP-PCR) are rapid and reliable diagnostic methods for the detection of *M. pneumoniae*; however, these methods are not widely available in Japanese hospitals, leading to a delay of several days to obtain results. Given that the rapid antigen test has a sensitivity of only 62.5% [5], *M. pneumoniae* infection cannot be ruled out based on a negative antigen test result. Consequently, treatment should begin based on clinical suspicion. Macrolides have been commonly used as the first-line agent for *M. pneumoniae* infection [6]. However, in East Asia, the incidence of macrolide-resistant *M. pneumoniae* (MRMP) is increasing [7]. Fluoroquinolones or tetracyclines are administered for MRMP infection [8]. Conversely, patients with severe CAP are often treated with a beta-lactam plus macrolide regimen based on the American Thoracic Society (ATS)/Infectious Diseases Society of America (IDSA) guideline recommendation [3,9]. Hence, if clinicians miss MRMP infection, initial treatment for severe CAP may fail [10]. Patients with severe *M. pneumoniae* pneumonia generally have low mortality rates; however, those admitted to the ICU exhibit a higher mortality rate [11]. Therefore, MRMP infection should not be ruled out in patients with high pre-test probability, even if the results of rapid antigen testing are negative and the patients have already received an initial macrolide-containing antimicrobial treatment. Here, we present the case of a young patient with severe MRMP pneumonia who required mechanical ventilation.

## Case presentation

**Figure 1:**
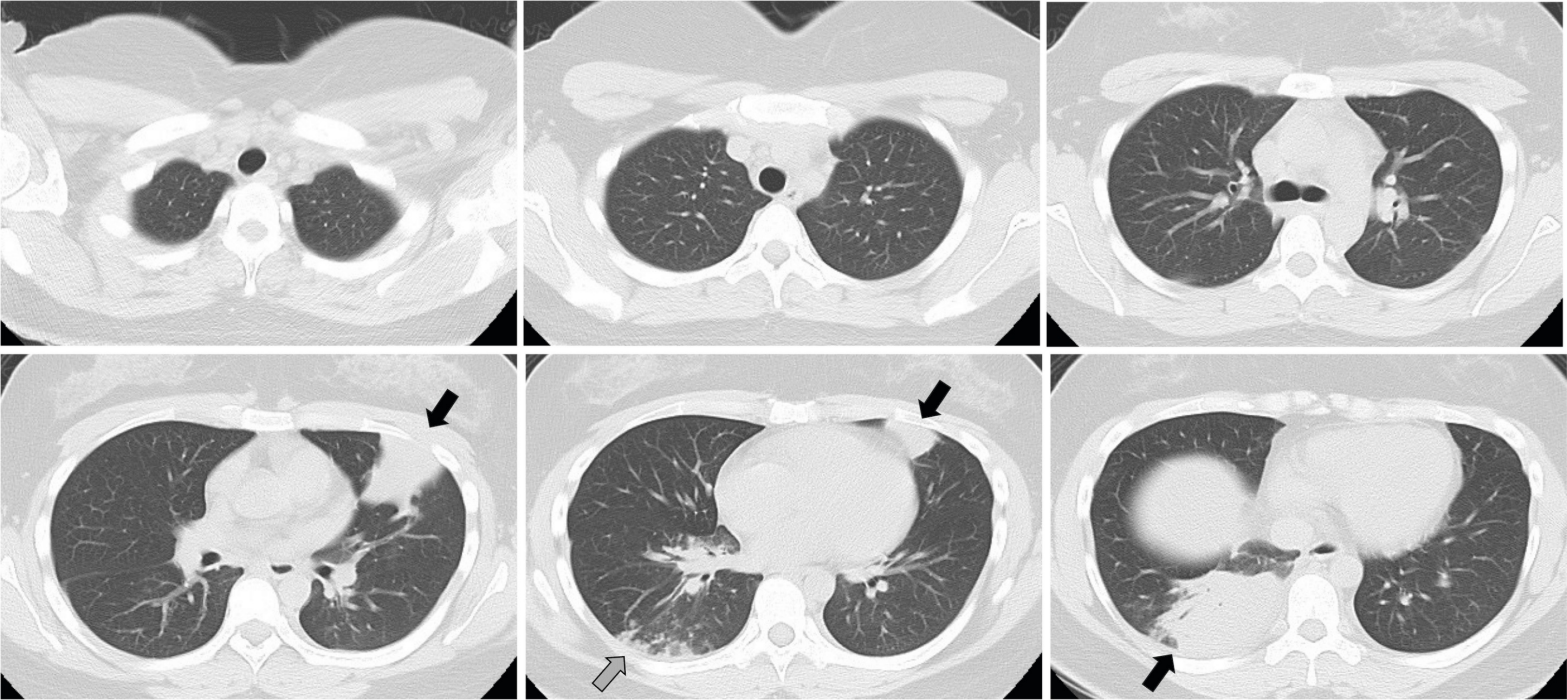
Chest computed tomography five days prior to admission showing centrilobular nodules, bronchial wall thickening (gray arrow), and consolidation in the right lower lobe and left lingular segment (black arrows)

A 27-year-old woman experiencing severe respiratory failure was transferred from another hospital to our ICU for further management. Five days before admission, she visited a local hospital with a four-day history of fever and cough. The patient had a history of depression and was taking milnacipran, amitriptyline hydrochloride, aripiprazole, flunitrazepam, etizolam, biperiden hydrochloride, and levomepromazine maleate. She worked as a clerk at a pharmacy. The patient did not consume alcohol or smoke. She had no pets, and none of her close contacts exhibited similar symptoms. Chest computed tomography (CT) revealed centrilobular nodules, bronchial wall thickening, and consolidation in the right lower lobe and left lingular segment (Figure 1). Rapid antigen tests for severe acute respiratory syndrome coronavirus 2 (SARS-CoV-2) and influenza A/B virus using nasopharyngeal swabs, *M. pneumoniae* using pharyngeal swabs, *Streptococcus pneumoniae*, and *Legionella pneumophila* using urine specimens were negative. The patient was diagnosed with CAP and received treatment with amoxicillin (500 mg)/clavulanate (125 mg) three times daily for five days and azithromycin (500 mg) once daily for three days. Although she took the medication as prescribed, her symptoms worsened. Therefore, on the day of ICU admission, she visited the local hospital presenting with severe respiratory failure, with an oxygen saturation of 92% on a 15-liter oxygen mask with a reservoir bag. Consequently, she was promptly intubated at an emergency department. After intubation, the partial pressure of arterial oxygen (PaO_2_) to inspired oxygen fraction (F_I_O_2_) ratio (P/F ratio) was 81 mmHg on an F_I_O_2_ of 1.0, with a positive end-expiratory pressure (PEEP) level of 12 cmH_2_O. Given the severity of her condition, venovenous extracorporeal membrane oxygenation (VV-ECMO) was considered, and she was subsequently transferred to the ICU facility at our hospital.

**Figure 2:**
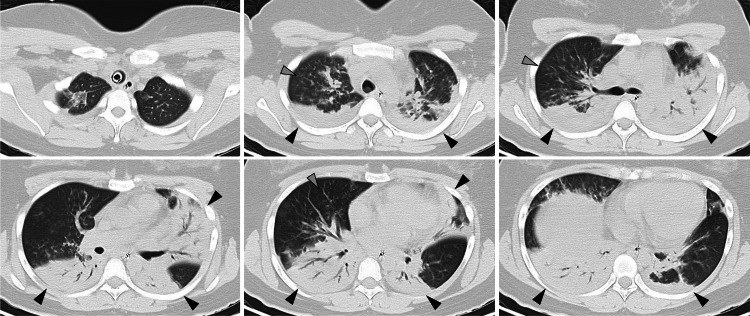
Chest computed tomography on the day of admission showing exacerbated bilateral consolidations (black arrowheads) and centrilobular nodules in the right lung (gray arrowheads)

Upon ICU admission, her vital signs were as follows: blood pressure, 98/59 mmHg; pulse rate, 104/min; body temperature, 37.7 ℃; and respiratory rate, 28 /min, with an oxygen saturation of 88% (F_I_O_2_ 1.0). The ventilator was set to pressure-controlled ventilation with an inspiratory pressure of 15 cm H_2_O, a ventilation rate of 20 breaths/min, and a PEEP level of 12 cm H_2_O. Arterial blood gas analysis showed a pH level of 7.326, a partial pressure of carbon dioxide (PaCO_2_) of 46.4 mmHg, a partial pressure of oxygen (PaO_2_) of 81.2 mmHg, and a bicarbonate level of 23.7 mmol/L. Laboratory tests showed elevated inflammatory markers and abnormal liver function results (Table 1). Serological tests for autoantibodies were negative (Table 2). The Nicking Enzyme Amplification Reaction (NEAR) test for SARS-CoV-2 and the repeat rapid antigen tests for influenza A/B virus,* M. pneumoniae*, *S. pneumoniae*, and *L. pneumophila* were all negative. A chest CT scan revealed exacerbation of consolidation, with an air bronchogram in the bilateral lungs and centrilobular nodules in the right lung (Figure 2). A transthoracic echocardiogram revealed a left ventricular ejection fraction of 60% with no valve disease.

**Table 1:** Laboratory findings on the day of ICU admission PT-INR, prothrombin time international normalized ratio; APTT, activated partial thromboplastin time; BNP, brain natriuretic peptide; KL-6, Krebs von den Lungen-6; HIV, human immunodeficiency virus.

Parameters	Patient’s lab values	Reference range
White blood cell count	10.8×10^3^/μL	3.3-8.6×10^3^/μL
Hemoglobin	9.1 g/dL	11.6-14.8 g/dL
Mean corpuscular volume	81.9 fL	83.6-98.2 fL
Platelet count	21.8×10^4^/μL	15.8-34.8×10^4^/μL
Serum sodium	140 mEq/L	138-145 mEq/L
Serum potassium	4.6 mEq/L	3.6-4.8 mEq/L
Urine nitrogen	10.5 mg/dL	8.0-20.0 mg/dL
Creatinine	0.74 mg/dL	0.46-0.79 mg/dL
Total bilirubin	0.4 mg/dL	0.4-1.5 mg/dL
Aspartate transaminase	36 U/L	13-30 U/L
Alanine transaminase	36 U/L	7-23 U/L
Alkaline phosphatase	67 U/L	38-113 U/L
Lactate dehydrogenase	352 U/L	124-222 U/L
Glucose	128 mg/dL	73-140 mg/dL
HbA1c	5.8 %	4.9-6.0 %
C-reactive protein	31.6 mg/dL	0.0-0.14 mg/dL
PT-INR	1.11	0.90-1.10
APTT	39.5 seconds	24.0-39.0 seconds
BNP	5.8 pg/mL	0.0-18.4 pg/mL
KL-6	175.0 U/mL	105.3-401.2 U/mL
IgG	953 mg/dL	861-1747 mg/dL
IgM	146 mg/dL	50-269 mg/dL
IgA	211 mg/dL	93-393 mg/dL
(1→3)-β-D-glucan	24.1 pg/mL	0.0-20.0 pg/mL
HIV antigen/antibody	negative	negative
Arterial blood gas		
pH	7.326	7.35-7.45
pO_2_ (F_I_O_2_: 1.0)	81.2	75-100 mmHg
pCO_2_	46.4	35-45 mmHg
Bicarbonate	23.7	21-31 mmol/L
Lactate acid	1.1	0.5-2.2 mmol/L

**Table 2:** Autoantibody test results on the day of ICU admission MPO-ANCA: myeloperoxidase anti-neutrophil cytoplasmic antibody, PR3-ANCA: proteinase3 anti-neutrophil cytoplasmic antibody, MDA-5: melanoma differentiation-associated gene 5, ARS: aminoacyl-tRNA synthetase, U1-RNP: uridin-1-ribonucleoprotein.

Autoantibodies	Patient’s lab values
Antinuclear antibody	Negative
MPO-ANCA	Negative
PR3-ANCA	Negative
Anti-SS-A antibody	Negative
Anti-SS-B antibody	Negative
Anti-Scl70 antibody	Negative
Anti-MDA-5 antibody	Negative
Anti-ARS antibody	Negative
Anti-U1-RNP antibody	Negative

The patient was diagnosed with severe ARDS caused by bilateral pneumonia. We performed bronchoalveolar lavage (BAL), and specimens were sent for bacterial cultures and genetic testing for *Chlamydophila pneumoniae*, *M. pneumoniae*, and *L. pneumoniae*. We administered levofloxacin (500 mg) once daily and vancomycin (1000 mg) every eight hours for severe pneumonia resistant to treatment with amoxicillin/clavulanate and azithromycin. Additionally, hydrocortisone (200 mg) was administered daily as a treatment for severe CAP. Concurrent with these treatments, we initiated a lung-protective ventilation strategy to manage severe ARDS. Although sedation with propofol and analgesia with fentanyl failed to control her strong spontaneous breathing effort, and the P/F ratio remained below 150 mmHg with a PEEP level of 12 cmH_2_O, we initiated continuous intravenous administration of rocuronium bromide along with prone position therapy. We measured the esophageal pressure and set a PEEP level of 16 cmH_2_O, ensuring a positive end-expiratory transpulmonary pressure. She was under volume-controlled ventilation with a tidal volume of 6.0 mL/kg predicted body weight, respiratory rate of 20 breaths/minute, plateau pressure of 28 cmH_2_O, and driving pressure of 12 cmH_2_O. Oxygenation improved after administering neuromuscular blockers and prone positioning therapy, and we determined that VV-ECMO was unnecessary. Two daily 16-hour prone sessions improved her P/F ratio to 360 mmHg at a PEEP level of 10 cmH_2_O. On the fourth day of hospitalization, the patient passed the spontaneous breathing trial and was successfully extubated. 

**Figure 3:**
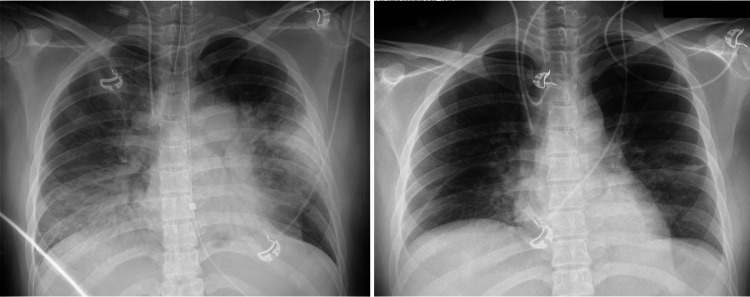
Chest radiography on day 1 and day 7 Chest radiography on day 1 (left side) revealed bilateral lung infiltration; however, on day 7 (right side), there was a significant improvement in the infiltration.

Based on the positive result of LAMP for *M. pneumoniae* and negative results from other tests (Table 3), the patient was diagnosed with *M. pneumoniae* pneumonia. On day 5, we discontinued vancomycin because of negative blood and sputum bacterial culture tests. Since we suspected MRMP clinically, we decided to continue levofloxacin, switching from intravenous to oral administration. Moreover, we terminated steroid treatment because the autoantibody results were negative, and the patient’s condition showed favorable improvement. On day 7, chest radiography showed improvement in bilateral infiltration (Figure 3). She was discharged unassisted on the 10th day. She was prescribed oral levofloxacin for 13 days, resulting in a total effective antibiotic course of 17 days. At her outpatient follow-up, one week after discharge, she reported no significant complications other than cough and completed her follow-up visit. 

**Table 3:** Results for causative agents BAL, bronchoalveolar lavage; LAMP, loop-mediated isothermal amplification; PA, particle agglutination.

Parameters	Patient’s lab values
BAL fluid	
Total cell count	2370/μL
Neutrocyte	61.0%
Lymphocyte	12.0%
Macrophage	27.0%
Eosinocyte	0.0%
Basocyte	0.0%
CD4^+^/CD8^+^ ratio	1.6
Legionella pneumophila LAMP	Negative
Pharyngeal swab test	
Mycoplasma pneumoniae LAMP	Positive
Serological test	
Mycoplasma pneumoniae	
antibody (PA) at day 1
antibody (PA) at day 14	×5120
Chlamydophila pneumoniae	
IgG antibody at day 1	4
IgG antibody at day 14	6

Eventually, *M. pneumoniae* was isolated within seven days using pleuropneumonia-like organisms (PPLO) medium in the BAL specimen. Additionally, the serum *M. pneumoniae *antibody level significantly increased two weeks post-admission. Genetic analysis revealed that the *M. pneumoniae* isolate had an A2063G transition, which is the most common mutation for macrolide resistance. The minimum inhibitory concentration (MIC) was measured using PPLO broth based on the broth microdilution protocol in the National Institute of Infectious Diseases (Japan). American Type Culture Collection type strains M129 and FH were used for quality control. The MIC was determined after 12 days of culture. The MIC of the isolate was 16 μg/mL for azithromycin and 0.25 μg/mL for levofloxacin (Table 4). The diagnosis of MRMP pneumonia was confirmed.

**Table 4:** MIC of M. pneumoniae isolate from the patient American Type Culture Collection type strains M129 and FH were used for quality control of the method. MLST, multilocus sequence typing; MIC, minimum inhibitory concentration.

	Patient	Control (M129)	Control (FH)
p1 Gene type	Type 1	Type 1	Type 2
MLST	ST3	ST1	ST2
Clade	T1-3R	T1-1	T2-B
MIC (μg/mL)			
Clarithromycin	128	< 0.12	<0.12
Azithromycin	8	<0.12	<0.12
Levofloxacin	0.25	0.25	0.25
Ofloxacin	0.5	1	0.5
Ciprofloxacin	0.5	0.5	0.25
Tosufloxacin	1	0.5	0.5
Minocycline	8	4	4
Tetracycline	2	0.5	0.5

## Discussion

A 27-year-old woman diagnosed with MRMP pneumonia with severe ARDS was discharged after 10 days of treatment with effective antibiotics, hydrocortisone, lung-protective ventilation, and prone positioning. 

In young adults, *M. pneumoniae* is a common pathogen of CAP [1], but it rarely leads to severe pneumonia [2,3]. In adult patients, the severity and rate of ICU admissions increase with age; the median age at ICU admission is 58.5 years ﻿[11]. There have been several reports of severe ARDS caused by *M. pneumoniae* pneumonia [3]; however, only one adult case of ARDS caused by MRMP pneumonia has been reported [10]. Despite her young age, the patient experienced rapid exacerbation and more severe respiratory failure. The exact mechanism of fulminant *M. pneumoniae* pneumonia remains unclear, making it difficult to predict which patients may develop severe conditions [2]. Although patients with *M. pneumoniae* pneumonia generally have low mortality rates, those admitted to the ICU exhibit a higher mortality rate [11]. Therefore, early diagnosis and effective treatment strategies are necessary.

Rapid and accurate diagnosis of *M. pneumoniae* pneumonia is often challenging. In this case, repeated rapid antigen tests for *M. pneumoniae* were negative. Rapid antigen testing is the most readily available test in Japan, detecting *M. pneumoniae *ribosomal protein L7/L12 using an immunochromatographic assay with results available within 15 minutes. However, its sensitivity of 62.5% and specificity of 90.9%, compared to the gold standard PCR [5], may lead to missed diagnosis. The most reliable diagnostic method for *M. pneumoniae* is a nucleic acid amplification test (NAAT), such as PCR. In Japan, LAMP and QP-PCR are common NAAT methods used in clinical practice, with results available within one hour. Although these tests are useful, obtaining results typically takes several days due to limited facilities that can perform in-hospital testing. Specific antibody tests in paired serum also take two to four weeks to confirm the diagnosis if the initial antibody level does not show a significant increase. In previous case reports of ARDS due to *M. pneumoniae* pneumonia, treatment was initiated based on the positive test results, including genetic tests [3,10]. As in this case, it is important to make an accurate clinical diagnosis in facilities that do not have access to such tests.

This case was clinically suspected to be *M. pneumoniae* pneumonia, based on the JRS scoring system and characteristic chest CT findings in the early phase. The JRS scoring system recommends that clinicians should strongly suspect *M. pneumoniae *pneumonia if five or more of the following six listed criteria are met: (1) age <60 years old, (2) no or mild co-morbidity, (3) obstinate cough, (4) poor findings from chest physical examination, (5) negative results on microbiological rapid diagnostic tests (except the positive result of antigen or genetic test for *M. pneumoniae*), and (6) white blood cell count < 10.0×10^3^/μL [12]. Here, the score at the initial visit was 6 points, and *M. pneumoniae* was strongly suspected. The radiographic features of *M. pneumoniae pneumonia* include bilateral bronchial wall thickening and centrilobular nodules [13]. In severe conditions, these findings progress to bilateral diffuse consolidation [13]. Strongly suspecting *M. pneumoniae* from images of patients presenting with severe ARDS is challenging.

Given that *M. pneumoniae* is not susceptible to beta-lactams, macrolides have been used as the first-line treatment for *M. pneumoniae* pneumonia [6]. However, macrolide resistance in *M. pneumoniae* has been rising in East Asia, with 11.3% to 65.3% of cases being macrolide-resistant in Japan and an alarming 88.3% of cases in a Chinese hospital [3,7]. In Japan, the A2063G mutation accounts for almost all (97.3-100%) macrolide-resistant mutations in the 23S rRNA gene of *M. pneumoniae* [7]. The isolate in this case also exhibited an A2063G transition mutation. The A2063G mutation is highly resistant to all macrolides but is susceptible to fluoroquinolones and tetracyclines [7]. The latest Japanese guideline classifies tetracyclines as first-line medications alongside macrolides for *M. pneumoniae *pneumonia owing to the high MRMP prevalence [12].

MRMP should be suspected when symptoms fail to improve within 48-72 hours of macrolide treatment [6,7]. In reported cases of severe *M. pneumoniae* pneumonia, the median onset of respiratory failure typically occurs 9-11.2 days (range, 3-21 days) after the initial symptoms appear [2,3]. Presently, respiratory failure developed nine days after the onset of symptoms. Close follow-up of patients who do not show improvement by day 3 of macrolide treatment may help in diagnosing MRMP and prevent the development of respiratory failure.

To confirm the diagnosis of MRMP promptly, QP-PCR is a useful technique to detect common macrolide resistance mutations. In Japan, GENECUBE®︎ and Smart Gene®︎ are commercially available medical diagnostic devices that detect mutations at positions 2063, 2064, 2067, and 2063, 2064 within domain V of the 23S rRNA gene, respectively. Recently, metagenomic next-generation sequencing (mNGS), a new testing method that analyzes genetic material (DNA and RNA) in patient samples, has shown its use in severe CAP. It can simultaneously detect broad-spectrum pathogens and resistance genes within 24 h, including MRMP. This study reported that mNGS of BAL fluid (BALF) in critically ill patients with pneumonia had a sensitivity of 95.3% and specificity of 63.1% in identifying the causative pathogens, with 61.2% of cases showing that the mNGS results positively influenced diagnosis and treatment [14]. Another study reported that mNGS of BALF for patients with severe pneumonia shortened the time to clinical improvement [15]. Although this method is costly and currently unavailable for clinical use in Japan, it could reduce the likelihood of overlooking MRMP.

The percentage of MRMP in *M. pneumoniae* changes periodically. The P1 gene of *M. pneumoniae* is divided into types 1 and 2. The isolate was type 1 lineage and is more often associated with drug-resistance genes than type 2 lineage [16]. A shift in lineage occurs approximately every decade in Japan; in 2019, before the coronavirus disease 2019 pandemic, the type 2 lineage was dominant and MRMP rates were declining [16]. In winter 2023, type 1 lineage became dominant again in China [17]. We should monitor its development as MRMP may increase again.

As for antimicrobial treatment for MRMP pneumonia, tetracyclines or fluoroquinolones are administered. A recent network meta-analysis showed that minocycline is the most effective agent in individuals over eight years of age and that moxifloxacin has greater advantages in effectiveness and safety than other fluoroquinolones such as levofloxacin [8]. However, a limited number of studies have explored the effectiveness of tetracyclines on *M. pneumoniae* pneumonia. Moreover, to our knowledge, there are no studies comparing the effectiveness of tetracyclines and fluoroquinolones in severe *M. pneumoniae* pneumonia. In the largest case series of severe *M. pneumoniae *pneumonia-induced ARDS from 2017 to 2019, all patients were treated with fluoroquinolones and had clinical improvement [3]. In a previous case report of severe MRMP pneumonia, treatment with levofloxacin and minocycline was initially combined [10]. However, there is insufficient evidence to support combination therapy, and this case suggests that monotherapy may be adequate. Further randomized controlled trials are needed to determine whether the use of tetracycline is superior in severely ill patients, such as the present case. To prevent antimicrobial resistance, tetracyclines may be preferable because M. pneumoniae has been shown to acquire fluoroquinolone resistance through genetic mutations in vitro [18].

Steroid administration is an optional therapy for severe *M. pneumoniae* pneumonia. *M. pneumoniae *can exacerbate pneumonia by producing the various inflammation-inducing factors that can activate host immune responses [2,19]. Based on immunopathological mechanisms, steroid administration may be beneficial in treating *M. pneumoniae* pneumonia [20]. Methylprednisolone, followed by prednisolone, is commonly chosen for the treatment of *M. pneumoniae* pneumonia, and there are few reports of hydrocortisone being used [13, 20]. A meta-analysis showed that glucocorticoid add-on treatment for MRMP shortened the duration of fever and hospital stay in children, and decreased CRP levels [20]. However, significant heterogeneity exists among studies, and the optimal dose and type of steroids required remain unknown. Additionally, it is unclear whether steroid use improves hard endpoints, such as mortality. In our case, we administered hydrocortisone at a dose of 200 mg daily based on the findings of a previous randomized controlled trial [4]. This study reported that early administration of low-dose hydrocortisone (200mg daily) reduced mortality in patients with severe CAP [4]. However, *M. pneumoniae* represented only 1.3% of the pathogens in the trial [4]. Hence, further studies are needed to determine whether corticosteroids, including hydrocortisone, can reduce mortality in severe *M. pneumoniae* pneumonia.

## Conclusions

Here, we present the case of a young patient with severe ARDS caused by MRMP. Clinicians should be aware that MRMP prevalence is high in East Asia, including Japan. For cases of severe pneumonia with a high pre-test probability of *M. pneumoniae*, MRMP should be considered, even if the rapid antigen test is negative and the patient is on macrolide treatment. Empiric treatment with tetracyclines or fluoroquinolones should be initiated until LAMP or QP-PCR results are available. Further studies on validating antimicrobial selections and steroid dosage regimens in severe cases are needed.
